# A retrospective evaluation of therapeutic efficacy and safety of chemoradiotherapy in older patients (aged ≥ 75 years) with limited-disease small cell lung cancer: insights from two institutions and review of the literature

**DOI:** 10.2478/raon-2024-0054

**Published:** 2024-09-15

**Authors:** Ayako Shiono, Hisao Imai, Satoshi Endo, Kazuki Katayama, Hideaki Sato, Kosuke Hashimoto, Yu Miura, Shohei Okazaki, Takanori Abe, Atsuto Mouri, Kyoichi Kaira, Ken Masubuchi, Kunihiko Kobayashi, Koichi Minato, Shingo Kato, Hiroshi Kagamu

**Affiliations:** Department of Respiratory Medicine, International Medical Center, Saitama Medical University, Saitama, Japan; Division of Respiratory Medicine, Gunma Prefectural Cancer Center, Gunma, Japan; Division of Radiation Oncology, Gunma Prefectural Cancer Center, Gunma, Japan; Department of Radiation Oncology, International Medical Center, Saitama Medical University, Saitama, Japan; Division of Health Evaluation and Promotion, SUBARU Health Insurance Society, Ota Memorial Hospital, Gunma, Japan

**Keywords:** chemoradiotherapy, chemotherapy, older patients, efficacy, limited disease, radiotherapy, safety, small cell lung cancer

## Abstract

**Background:**

The standard treatment for patients in good general condition with limited-disease small cell lung cancer (LD-SCLC) is concurrent platinum/etoposide chemotherapy and thoracic radiotherapy (TRT). However, the efficacy and safety of chemoradiotherapy (CRT) in older patients with LD-SCLC has not been fully explored; moreover, the optimal treatment for this patient group remains unclear. This study aimed to investigate the feasibility and efficacy of CRT in older patients with LD-SCLC.

**Patients and methods:**

From April 2007 to June 2021, consecutive older patients (aged ≥ 75 years) with stage I to III SCLC who received concurrent or sequential CRT at two institutions were retrospectively evaluated for efficacy and toxicity of CRT.

**Results:**

A total of 32 older patients underwent concurrent (n = 19) or sequential (n = 13) CRT for LD-SCLC. The median ages of the patients in the concurrent and sequential CRT groups were 77 (range: 75–81) years and 79 (range: 76–92) years, respectively. The median number of chemotherapeutic treatment cycles was four (range, 1–5), and the response rate was 96.9% in all patients (94.7% in concurrent and 100% in sequential CRT groups). The median progression-free survival (PFS) and median overall survival (OS) for all patients were 11.9 and 21.1 months, respectively. The median PFS was 13.0 and 9.0 months in the concurrent CRT and sequential CRT groups, respectively, with no statistically significant difference (*p* = 0.67). The median OS from the initiation of CRT was 19.2 and 23.5 months in the concurrent and sequential CRT groups, respectively (*p* = 0.46). The frequencies of Grade ≥ 3 hematological adverse events were as follows: decreased white blood cell count, 20/32 (62.5%); decreased neutrophil count, 23/32 (71.9%); anemia, 6/32 (18.8%); decreased platelet count, 7/32 (21.9%); and febrile neutropenia, 3/32 (9.4%). Treatment-related deaths occurred in one patient from each group.

**Conclusions:**

Although hematological toxicities, particularly reduced neutrophil count, were severe, CRT showed favorable efficacy in both concurrent and sequential CRT groups. However, concurrent CRT may not be feasible for all older patients with LD-SCLC; accordingly, sequential CRT may be considered as a treatment of choice for these patients. Further prospective trials are warranted to identify optimal treatment strategies for this patient group.

## Introduction

Lung cancer is the leading cause of cancer-related deaths worldwide.^1^ Small cell lung cancer (SCLC) accounts for 10–15% of all lung cancers and is an aggressive tumor characterized by early development of extensive metastases and rapid growth.^2,3^ Limited-disease SCLC (LD-SCLC) is restricted to one hemithorax and its regional lymph nodes, and it can be treated with a single radiotherapy field. Furthermore, LD-SCLC accounts for one-third of all SCLCs cases at the time of diagnosis.^1^ The proportion of older patients with SCLC continues to increase with the growing geriatric population.^4,5^ Approximately 30–40% of patients with SCLC are ≥ 70-years-old at their diagnosis^6^, and it is becoming increasingly crucial to understand how SCLC therapy should be tailored for older patients.

The standard treatment for patients with LD-SCLC in good general condition is concurrent platinum/etoposide chemotherapy and thoracic radiotherapy (TRT), followed by prophylactic cranial irradiation (PCI) for those who respond to chemoradiotherapy (CRT).^7,8^ However, many clinical studies on LD-SCLC have precluded the enrollment of older patients for reasons such as a decline in organ function or comorbidities.^9,10^ For example, a previous study demonstrated that a cisplatin plus etoposide combination regimen and concurrent TRT are more effective for the treatment of LD-SCLC than a cisplatin plus etoposide combination and sequential TRT^11^; however, it is noteworthy that patients aged ≥ 75 years were precluded from enrolling in the study.

Retrospective subset studies of patients with LD-SCLC treated with cisplatin, along with etoposide and concurrent early CRT, in randomized phase III studies have demonstrated that severe hematological adverse event, pneumonitis of Grade 4 or more, and treatment-related deaths were observed more frequently in older patients aged ≥ 70 years than their younger counterparts.^12,13^ Although the objective response rate and 5-year event-free survival rate were not significantly different between these two subgroups, there was a trend for them to be worse in older patients. Notably, a significant difference in the 5-year overall survival rate was observed in patients < 70 years of age in one trial.^12,13^ These results imply that the combination of cisplatin and etoposide is toxic to older patients with LD-SCLC, and that the most suitable treatment remains unclear.

However, the therapeutic efficacy and toxicity of CRT in older patients with LD-SCLC have not yet been fully examined. In particular, as mentioned above, older patients with LD-SCLC aged ≥ 75 years are excluded from clinical trials^11^ or studies focusing on patients aged ≥ 75 years are scarce. Thus, the aim of our analysis was to retrospectively evaluate the safety and treatment efficacy of CRT and to explore the most suitable therapy for older patients with LD-SCLC aged ≥ 75 years. We assessed patient backgrounds, treatment compliance, treatment efficacy, and toxicity between patients who underwent concurrent and sequential CRT.

## Patients and methods

### Patients

We retrospectively analyzed the medical records of consecutive patients with Stage I–III LD-SCLC, aged ≥ 75 years, whose treatment plan involved concurrent or sequential CRT between April 2007 and June 2021 at two Japanese institutions (International Medical Center, Saitama Medical University and Gunma Prefectural Cancer Center). The requirement for written informed consent was waived by the Ethics Committee of Saitama Medical University owing to the retrospective nature of the study. All procedures complied with the tenets of the Declaration of Helsinki. The study design was approved by the Institutional Ethics Committee of the International Medical Center at Saitama Medical University (approval number 2023-033).

The inclusion criteria were as follows: (i) older patients aged ≥ 75 years with cytologically or histologically diagnosed SCLC; (ii) patients with involvement of one hemithorax and its regional lymph nodes that could be treated with a single radiotherapy field; and (iii) patients that underwent first-line CRT (concurrent or sequential). The clinical stage of SCLC was determined based on the Union for International Cancer Control tumor-node-metastasis (TNM) Classification, Seventh Edition.^14^ The inclusion criteria for concurrent or sequential CRT at our institutions are as follows: patients with a performance status (PS) of 0–2; neutrophil count, ≥ 1.5 × 10^3^/mm^3^; platelet count, ≥ 1.0 × 10^5^/mm^3^; serum creatinine, ≤ 1.5 mg/dl; total bilirubin, ≤ 2.0 mg/dl; and a transaminase level ≤ 100 U/L.

All patients underwent pretreatment physical examinations, chest radiography, computed tomography (CT) scans of the chest/abdomen, CT or magnetic resonance imaging of the brain, and bone scintigraphy/^18^F-fluorodeoxyglucose positron-emission tomography to assess the TNM disease stage. Data of each patient were extracted from the electronic medical records.

### Treatment

#### Chemotherapy

A combination of etoposide (60–100 mg/m^2^) on days 1–3 plus cisplatin (60–80 mg/m^2^) on day 1 or carboplatin (area under the curve [AUC] 3–5) on day 1 was administered intravenously every 3–4 weeks. The chemotherapeutic agent and its dose were determined by an attending physician. The chemotherapeutic administration cycles were repeated every 3–4 weeks. At our institution, the criteria for initiating subsequent cycles of chemotherapy were the same as the criteria for the inclusion of concurrent or sequential CRT as described in the Patient subsection. If these criteria were not met, subsequent cycles were withheld until the dosing criteria were met. If the dosing criteria were not met seven weeks after the first day of the cycle, chemotherapy was discontinued. Generally, the doses of etoposide and platinum (cisplatin or carboplatin) are reduced or chemotherapeutic regimens are altered in the adverse event of Grade 4 decreased platelet count, prolonged Grade 4 decreased white blood cell count / decreased neutrophil count, or Grade 3 or more severe non-hematological toxicity during the previous chemotherapeutic cycle. For neutropenia, a granulocyte colony-stimulating factor was administered as prophylaxis at the discretion of the attending physician. Treatment was terminated when disease progression was observed, intolerable toxicity occurred, or when the patient withdrew consent for treatment.

#### Radiotherapy

Generally, TRT is started concurrently in the first cycle of chemotherapy or sequentially after four cycles of chemotherapy in older patients with LD-SCLC. The prescribed dose was 45 Gy in 30 fractions (1.5 Gy twice-daily) for the concurrent case and 60 Gy in 30 fractions (2 Gy daily) for the sequential case. All the patients underwent chest CT to facilitate treatment planning. The primary tumor (gross tumor volume [GTV] primary) was delineated in the pulmonary windows, and nodal involvement (GTV node) was delineated in the mediastinal windows. A clinical target volume (CTV) margin of 5 mm was added to the GTV primary and node. To plan the target volume margin, 5 mm was added to the CTV to ensure that the dose reached the target volume. The initial field in the sequential arm was based on pretreatment tumor volume. Regarding dose constraints, for normal lung volume receiving > 20 Gy (V20), the dose was ≤ 35% of the total lung volume and maximum spinal cord dose was < 45 Gy in a once-daily fraction regimen or < 36 Gy in twice-daily fractions regimen. Additionally, TRT was suspended if the patient experienced a decrease in Grade 4 platelet count, radiation pneumonitis, fever caused by infection, decrease in arterial oxygen pressure exceeding 10 mmHg, or if the patient had difficulty swallowing a liquid diet.

After TRT, PCI was administered to patients with a complete or near-complete response represented by a scar-like shadow on chest CT if the physician in charge judged that the patient would benefit from PCI, which consisted of 25 Gy/10 fractions for the entire brain.

### Evaluation of treatment response and adverse events

The best overall response and maximum tumor shrinkage were evaluated as tumor responses. Radiographic tumor responses were classified based on the Response Evaluation Criteria in Solid Tumors (RECIST), version 1.1.^15^ Tumor responses were defined as complete response (CR), partial response (PR), stable disease (SD), progressive disease (PD), or not evaluated (NE). If treatment failure occurred, the patients were permitted any subsequent treatment based on their preferences. Treatment CRT-related adverse events were graded according to the Common Terminology Criteria for Adverse Events (version 4.0).

### Statistical analysis

Categorical variables were analyzed using Fisher’s exact test, and continuous variables were analyzed using Welch’s t-test. Progression-free survival (PFS) was calculated from the start of treatment until PD or death from any cause, and overall survival (OS) was calculated from the first day of treatment until death or censored on the date of the last follow-up. Survival curves were calculated using the Kaplan–Meier method and compared between the two groups using the log-rank test. Differences were considered statistically significant at a two-tailed *p*-value of < 0.05. All statistical analyses were performed using the JMP statistical software, version 11.0, for Windows (SAS Institute, Cary, NC, USA).

## Results

### Patient characteristics

The patient selection process is illustrated in Supplementary Figure 1. Thirty-two patients were treated with CRT between April 2007 and June 2021 at both institutions (concurrent CRT group, n = 19; sequential CRT group, n = 13) and were assessed for response, survival, and safety of the treatments. Table 1 shows the patient characteristics in the concurrent/sequential CRT group. Men comprised a majority of the patients (n = 27, 84.3%), and the median age of the entire group was 78 (range, 75–92) years. A total of 96.8% of patients had a PS of 0 or 1, and the remaining patients had a PS of 2. All the patients were smokers, and 71.8% had a disease stage of III. No significant differences were observed in the baseline patient characteristics between the concurrent and sequential CRT groups. The median number of chemotherapeutic treatment cycles was four (range 1–4) in the concurrent CRT group and four (range 2–5) in the sequential CRT group.

**TABLE 1.** Baseline patient characteristics

**Characteristic**	**Total (*N* = 32)**	**Concurrent CRT group (*n* = 19)**	**Sequential CRT group (*n* = 13)**	***p* ^a^**
**Sex**				
Male / female	27 / 5	16 / 3	11 / 2	> 0.99
**Age (years)**				
Median	78	77	79	0.05^b^
Range	75–92	75–81	76–92	
**ECOG-PS, *n***				
0 / 1 / 2 / 3 / 4	12 / 19 / 1 / 0 / 0	6 / 12 / 1 / 0 / 0	6 / 7 / 0 / 0 / 0	
**Smoking status, *n***				
Yes / no	32 / 0	19 / 0	13 / 0	> 0.99
**Histology, *n***				
Small cell carcinoma / combined small cell carcinoma	29 / 3	18 / 1	11 / 2	0.55
**Disease stage, *n***				
I / II / III / postoperative recurrence	5 / 4 / 23 / 0	4 / 2 / 13 / 0	1 / 2 / 10 / 0	
**History of postoperative adjuvant chemotherapy, *n***				
Yes / no	0 / 32	0 / 19	0 / 13	> 0.99
**Number of cycles chemotherapy administered, *n***				
Median	4	4	4	0.19b
Range	1–5	1–4	2–5	
**Chemotherapy regimen, *n***				
CBDCA+etoposide / CDDP+etoposide	28 / 4	16 / 3	12 /1	0.63
**With or without G-CSF prophylaxis, *n***				
Yes / no	27 / 5	17 / 2	10 / 3	0.37
**Radiation irradiation method, *n***				
Conventional / accelerated hyperfractionated radiotherapy	26 / 6	14 / 5	12 / 1	0.36
**Completion of chemotherapy, *n***				
Yes / no	21 / 11	11 / 8	10 / 3	0.45
**Completion of radiotherapy, *n***				
Yes / no	31 / 1	18 / 1	13 / 0	> 0.99
**Prophylactic cranial irradiation, *n***				
Yes / no	2 / 30	2 / 17	0 / 13	0.50
**Reason for discontinuation of chemotherapy administration^b^, *n***				
Progressive disease	1^c^	0		
Adverse events	7	6	1	
Others	3	2	1	
**Alive at data cutoff, *n***				
Alive / death	8 / 24	4 / 15	4 / 9	0.68

CBDCA = carboplatin; CDDP = cisplatin; CRT = chemoradiotherapy; ECOG-PS = Eastern Cooperative Oncology Group - Performance Status; G-CSF = granulocyte colony-stimulating factor

a Comparison between the concurrent and sequential chemoradiotherapy groups

b Welch’s *t*-test

c The clinical progressive disease after two courses of chemotherapy, followed by definitive radiotherapy and partial response (PR)

Most patients (28, 87.5%) were treated with carboplatin and etoposide in combination with radiotherapy. Supplementary Table 1 lists the treatment delivery. The most frequently administered doses in the concurrent CRT group were AUC 4 for carboplatin and 80 mg/m^2^ for etoposide (n = 9 patients, 47.3%), and in the sequential CRT group, they were AUC 5 for carboplatin and 80 mg/m^2^ for etoposide (n = 4 patients, 30.7%).

### Treatment response and survival

Table 2 shows the results of the treatment response. The response rate was 94.7% in the concurrent CRT group (CR, n = 3; PR, n = 15; SD, n = 0; and PD, n = 0) and 100.0% in the sequential CRT group (CR, n = 0; PR, n = 13; SD, n = 0; and PD, n = 0). No significant differences in treatment response were observed between the concurrent and sequential CRT groups.

**TABLE 2.** Treatment response

**Response**	**Total (*N* = 32)**	**Concurrent CRT (*n* = 19)**	**Sequential CRT (*n* = 13)**	***p* ^a^**
Complete response	3	3	0	
Partial response	28	15	13	
Stable disease	0	0	0	
Progressive disease	0	0	0	
Not evaluated	1	1	0	
**Response rate (%) (95% CI)**	96.9 (82.9–100)	94.7 (73.5–100)	100 (−)	> 0.99
**Disease control rate (%) (95% CI)**	96.9 (82.9–100)	94.7 (73.5–100)	100 (−)	> 0.99

CRT = chemoradiotherapy; 95% CI = 95% confidence interval

a Comparison between the concurrent and sequential chemoradiotherapy groups

Regarding survival, median PFS was 11.9 (95% CI: 8.2–15.2) months (Figure 1A) and median OS was 21.1 (95% CI: 13.0–39.5) months (Figure 1B) for all patients. No significant differences were observed in the PFS or OS between concurrent and sequential CRT groups. Median PFS was 13.0 (95% CI: 7.8–18.2) months in the concurrent group and 9.0 (95% CI: 6.0–not reached) months in the sequential group (*p* = 0.67; Figure 2A). Median OS was 19.2 (95% CI: 11.0–37.1) months in the concurrent CRT group and 23.5 (95% CI: 11.0–not reached) months in the sequential CRT group (*p* = 0.46; Figure 2B).

**FIGURE 1A.**
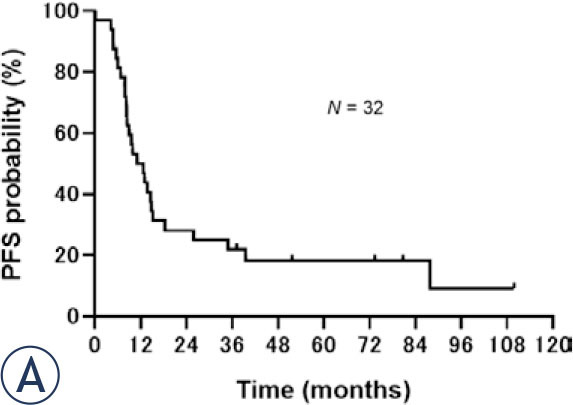
Kaplan-Meier analysis of the progression-free survival of the 32 patients. The median progression-free survival was 11.9 months.

**FIGURE 1B.**
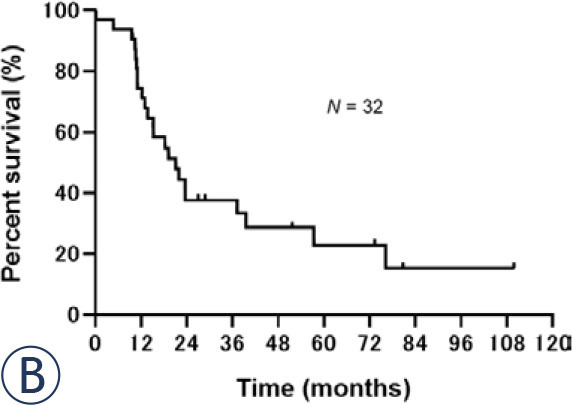
Kaplan-Meier analysis of the overall survival of 32 patients. The median overall survival was 21.1 months.

**FIGURE 2A.**
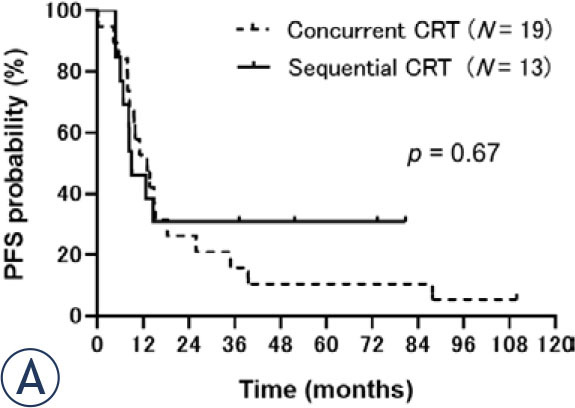
Progression-free survival (PFS) of the concurrent and sequential chemoradiotherapy groups. The median PFS was 13.0 months in the concurrent group and 9.0 months in the sequential group (*p* = 0.67).

**FIGURE 2B.**
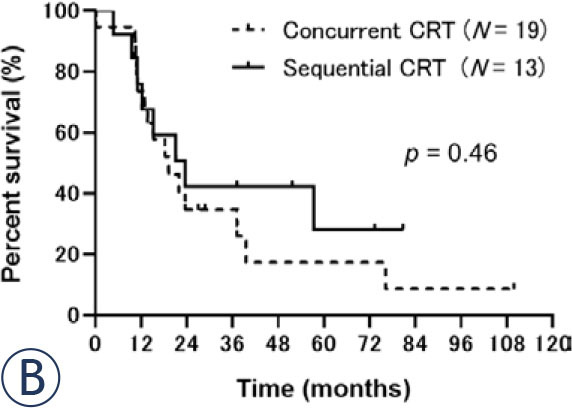
The overall survival (OS) of the concurrent and sequential chemoradiotherapy groups. The median OS was 19.2 months in the concurrent group and 23.5 months in the sequential group (*p* = 0.46).

### Toxicity

Treatment-related adverse events of all the patients are presented in Table 3. Toxicity was evaluated in all 32 patients. Myelosuppression was the most frequent treatment-related adverse event—decreased neutrophil counts (Grade 3 or 4) were seen in 71.9% patients and decreased white blood cell counts (Grade 3 or 4) in 62.5% patients. Febrile neutropenia was observed in three patients (9.4%). Grade 3 or 4 anemia occurred in six patients (18.8%), and decreased platelet counts (Grade 3 or 4) in seven patients (21.9%). The incidence of non-hematological toxicities was low, and the most frequent Grade 3 or 4 non-hematologic toxicity was infection (12.5%). Grade 3 or 4 pneumonitis was seen in two patients. Adverse events leading to treatment discontinuation occurred in 6/19 (31.6%) patients in the concurrent CRT group and 1/13 (7.7%) patients in the sequential CRT group and were more frequent in the concurrent CRT group, although the difference was not significant (*p* = 0.06). Treatment-related deaths occurred in two patients, one in each group. One patient suffered from pneumonitis in the sequential group and another patient suffered from acute coronary syndrome in the concurrent group.

**TABLE 3.** Adverse events

**Adverse event**	**All patients (*N* = 32)**				**Concurrent CRT (*n* = 19)**				**Sequential CRT (*n* = 13)**				***p* ^a^**

	**Any Grade**	**%**	**Grade≥3**	**%**	**Any Grade**	**%**	**Grade≥3**	**%**	**Any Grade**	**%**	**Grade≥3**	**%**	
**Led to discontinuation**	7	21.9	6	18.8	6	31.6	6	31.6	1	7.7	0	0.0	0.06
**Led to death**	-		2	6.3	-	-	1	5.3	-	-	1	7.7	> 0.99
**Treatment related^b^**													
White blood cell decreased	28	87.5	20	62.5	18	94.7	16	84.2	10	76.9	4	30.8	**0.004**
Neutrophil count decreased	26	81.3	23	71.9	18	94.7	15	78.9	8	61.5	8	61.5	0.43
Anemia	28	87.5	6	18.8	16	84.2	5	26.3	12	92.3	1	7.7	0.36
Platelet count decreased	27	84.4	7	21.9	16	84.2	5	26.3	11	84.6	2	15.4	0.67
Febrile neutropenia	3	9.4	3	9.4	2	10.5	2	10.5	1	7.7	1	7.7	> 0.99
Diarrhea	3	9.4	1	3.1	2	10.5	1	5.3	1	7.7	0	0.0	> 0.99
Constipation	14	43.8	1	3.1	9	47.4	0	0.0	5	38.5	1	7.7	0.41
Dermatitis radiation	8	25.0	1	3.1	2	10.5	1	5.3	6	46.2	0	0.0	> 0.99
Pneumonitis	29	90.6	2	6.3	18	94.7	1	5.3	11	84.6	1	7.7	> 0.99
Infection	7	21.9	4	12.5	4	21.1	3	15.8	3	23.1	1	7.7	0.63
Pneumothorax	2	6.3	2	6.3	2	10.5	2	10.5	0	0.0	0	0.0	0.50
Hypotension	1	3.1	1	3.1	1	5.3	1	5.3	0	0.0	0	0.0	> 0.99
Generalized muscle weakness	1	3.1	1	3.1	1	5.3	1	5.3	0	0.0	0	0.0	> 0.99
Acute coronary syndrome	1	3.1	1	3.1	1	5.3	1	5.3	0	0.0	0	0.0	> 0.99

CRT = chemoradiotherapy. Bold text indicates statistically significant differences.

a Comparison between the concurrent cohort and sequential chemoradiotherapy groups of Grade ≥ 3.

b Treatment-related adverse events reported as Grade ≥ 3 in ≥ one patient.

Analysis of myelosuppression revealed that hematological toxicities occurring with sequential CRT were milder than those with concurrent CRT (Table 3). The frequencies of Grade 3 or 4 hematologic toxicities in patients receiving sequential CRT versus those receiving concurrent CRT were as follows: white blood cell count decreased by 30.8% versus 84.2%, respectively (*p* = 0.004); neutrophil count decreased by 61.5% versus 78.9%, respectively (*p* = 0.43); anemia decreased by 7.7% versus 26.3%, respectively (*p* = 0.36); and platelet count decreased by 15.4% versus 26.3%, respectively (*p* = 0.67). Febrile neutropenia occurred in 7.7% of patients receiving sequential CRT and in 10.5% of patients receiving concurrent CRT. Other non-hematologic toxicities, such as Grade 3 or higher diarrhea, dermatitis, radiation, infection, pneumothorax, hypotension, generalized muscle weakness, and acute coronary syndrome, were more common in the concurrent CRT group; however, this was not statistically significant.

### Subsequent treatment after CRT

Subsequent treatment administered after CRT is presented in Table 4 and recurrence was observed in 27/32 patients. The best supportive care was often the treatment of choice for patients with recurrence after CRT, with a post-relapse chemotherapy conversion rate of 13/27 (48.1%) patients. The most common subsequent chemotherapy was a combination of carboplatin and etoposide, followed by amrubicin monotherapy. Six patients received up to third-line treatment; however, no patients received chemotherapy beyond the fourth-line treatment.

**TABLE 4.** Overview of subsequent chemoradiotherapy treatments

	**Second-line**	**Third-line**	**≥ Fourth-line**
Carboplatin+etoposide	7	0	0
Carboplatin+etoposide+atezolizumab/durvalmab	2	0	0
Carboplatin+irinotecan	0	1	0
Carboplatin+paclitaxel	0	1	0
Amurubicin	4	2	0
Nogitecan	0	0	0
Irinotecan	0	0	0
Others	0	2	0
Best supportive care	14	-	-
No recurrence	5		

## Discussion

This retrospective study assessed the efficacy and safety of CRT in older patients with LD-SCLC. Concurrent and sequential CRT groups demonstrated similar efficacy in the treatment of older patients with LD-SCLC; however, the toxicity profiles tended to be higher in the concurrent CRT group. These safety profiles should be considered when using CRT to treat older patients with LD-SCLC.

Meta-analyses and prospective and retrospective studies specifically focused on older patients with LD-SCLC have shown conflicting results regarding the survival benefits and tolerability of CRT.^16,17,18,19,20,21,22,23,24,25,26,27^ In the CONVERT trial, Christodoulou *et al*. reported the treatment outcomes of a subgroup of patients aged ≥ 70 years with LD-SCLC compared to those of younger patients.^26^ Concurrent CRT was found to be feasible in selected, fit older patients with LD-SCLC. Findings of previous studies on CRT in older patients with LD-SCLC are summarized in Table 5, along with our findings. Considering the findings of previous prospective trials evaluating CRT in older patients (≥ 70 years) with LD-SCLC, we infer that the response rate, PFS, and OS obtained in our study were satisfactory.^17,23,24,26,27^ In meta-analyses and prospective and retrospective studies of older patients with LD-SCLC, the response rates in both the concurrent and sequential CRT groups generally ranged from 70–100%, with PFS ranging from 9–14 months and OS from 17–29 months. Moreover, the therapeutic efficacies were similar, except for those reported by Jeremic *et al*. and Corso *et al.* in which the OS was 15 months and 15.6 months, respectively.

**TABLE 5.** Findings of previous studies on chemoradiotherapy in older patients with limited-disease small cell lung cancer

**Report [ref]**	**Year**	**Region**	**Age (years)**	**Study type**	**Sample size**	**PS**	**Stage**	**Treatment**	**Response rate (%) (All, con CRT vs. seq CRT)**	**PFS (months) (All, con CRT vs. seq CRT)**	**OS (months) (All, con CRT vs. seq CRT)**	**Interruption of treatment**	**Grade 3 or higher^a^**
Jeremic *et al*.^17^	1998	Yugoslavia	≥ 70	Prospective, Phase 2	72	KPS≥60	Limited disease	concurrent CRT (CBDCA+ETP)	75	NR	15	NR	Leukopenia 8.3%, Thrombocytopenia 11%, Infection 4.2%, Pneumonitis 18%
Shimizu *et al*.^18^	2007	Japan	≥ 75	Retrospective	7	0–1	II–III	concurrent CRT (CBDCA+ETP or CDDP+ETP)	100	NR	24.7	Imcompleted intent cycles of chemotherapy 3/7 (42.8%)	Leukopenia 100%, Neutropenia 100%, Thrombocytopenia 57.1%, FN 42.8%, Pneumonitis 28.5%
Okamoto *et al*.^19^	2010	Japan	≥ 70	Retrospective	12	0–1	II–III	concurrent CRT (CDDP+ETP)	100	14.2	24.1	Imcompleted intent cycles of chemotherapy 5/12 (41.7%)	Leukopenia 100%, Neutropenia 100%, Thrombocytopenia 33%, FN 67%, Pneumonitis 8%
Shukuya *et al*.^20^	2013	Japan	≥ 75	Retrospective	20	0–1	II–III	concurrent CRT (CBDCA+ETP or CDDP+ETP); n=5, sequential CRT (CBDCA+ETP or CDDP+ETP); n=15	NR, 100 *vs.* 80	NR, 208 days *vs.* 216 days	601 days (seq CRT with CBDCA+ETP)	Con *vs.* seq CRT; Imcompleted intent cycles of chemotherapy 2/5 (40%) *vs.*, 2/15 (13.3%)	Con *vs.* seq CRT; Leukopenia 100% *vs.* 53%, Neutropenia 100% *vs.* 93%, Thrombocytopenia 20% *vs.* 27%, FN 60% *vs.* 13%, Infection 0% *vs.* 7%, Pneumonitis 0% *vs.* 27%
Okamoto *et al*.^21^	2014	Japan	≥ 70	Prospective, Phase 1	12	0–1	Limited disease	concurrent CRT (split CDDP+ETP)	91.6	11.5	17	Imcompleted intent cycles of chemotherapy 5/12 (41.6%)	Leukopenia 100%, Neutropenia 100%, Thrombocytopenia 33%, FN 33%, Pneumonitis 16% (level 2 cohort)
Corso *et al*.^22^	2015	U.S.A	≥ 70	Retrospective	4362^b^	NR	I–III	concurrent CRT; n=3472, sequential CRT; n=1136	NR	NR	15.6, 17.0 *vs.* 15.4	NR	NR
Kubo *et al*.^23^	2016	Japan	≥ 76	Prospective, Phase 2	22	0–2	I–III	sequential CRT (CDDP+TOP)	68	9.1	22.2	Imcompleted intent treatment course of CRT 41%	Neutropenia 96%, Thrombocytopenia 50%, FN 32%, Pneumonitis 18%
Misumi *et al*.^24^	2017	Japan	≥ 70	Prospective, Phase 1/2	35^c^	0–2	I–III	sequential CRT (CBDCA+CPT11)	88.6	11.2	27.1	Imcompleted intent cycles of chemotherapy 7/35 (20.0%)	Neutrophils 51%, Platelets 11.4%, FN 5.7%, Pneumonitis 5.7%
Stinchcombe *et al*.^25^	2019	USA	≥ 70	Pooled analysis	254	NR	Limited disease	concurrent CRT (CBDCA+ETP or CDDP+ETP)	NR	10.6	17.8	Imcompleted intent treatment course of CRT	Neutropenia 56%, Pneumonitis 2%
Christodoulou *et al*.^26^	2019	Europe	≥ 70	Prospective, Phase 3 (subgroup)	67	0–2	I–III	concurrent CRT (CDDP+ETP)	NR	18	29	135/250 (54%) Imcompleted intent cycles of chemotherapy 25/67 (37.3%)	Neutropenia 84%, Thrombocytopenia 28%, Infection 13%, Pneumonitis 3%
Killingberg *et al.*^27^	2023	Norway	≥ 70	Prospective, Phase 2 (subgroup)	53	0–2	I–III	concurrent CRT (CBDCA+ETP or CDDP+ETP)	70	12.2	24	Imcompleted TRT as planned 8%, Imcompleted four cycles of chemotherapy 15%	Neutropenia 80%, Thrombocytopenia 30%, Infection 4%, Pneumonitis 4%
Current study		Japan	≥ 75	Retrospective	32	0–2	I–III	concurrent CRT (CBDCA+ETP or CDDP+ETP); n=19, sequential CRT (CBDCA+ETP or CDDP+ETP); n=13	96.9, 94.7 *vs.* 100	11.8, 13.0 *vs.* 9.0	21.1, 19.2 *vs.* 23.5	Con *vs.* seq CRT; Imcompleted intent cycles of chemotherapy 6/19 (31.6%) *vs.* 1/13 (7.7%)	Con *vs.* seq CRT; White blood cell decreased 84.2% *vs.* 30.8%, Neutrophil count decreased 78.9% *vs.* 61.5%, Platelet count decreased 26.3% *vs.* 15.4%, FN 10.5% *vs.* 7.7%, Infection 15.8% *vs.* 7.7%, Pneumonitis 5.3% *vs.* 7.7%

CBDCA = carboplatin; CDDP = cisplatin; con CRT = concurrent chemoradiotherapy; CPT11 = irinotecan; ETP = etoposide; FN = febrile neutropenia; KPS = Karnofsky performance status; NR = not reported; OS = overall survival; PFS = progression-free survival; PS = performance status; seq CRT = sequential chemoradiotherapy

a Some studies use different versions of the Common Terminology Criteria for Adverse Events.

b Patients receiving CRT with survival of at least 4 months after diagnosis

c Phase 2 cohort

To the best of our knowledge, only a few studies on CRT have been conducted to date in older patients aged ≥ 70 years, and only three studies have been conducted on patients aged ≥ 75 years (two retrospective and one prospective study^18,20,23^, both with small numbers of cases; Table 5). A comparison of toxicities between concurrent and sequential CRT groups showed that the frequency of Grade 3 or higher myelosuppression (particularly leukopenia) was higher in the concurrent than in sequential CRT group. However, Kubo *et al*. reported that in the sequential CRT group, Grade 3 or higher neutropenia, thrombocytopenia, febrile neutropenia, and pneumonia were relatively common, which may have been influenced by the chemotherapeutic regimen of cisplatin and topotecan therapy.^23^ In general, except in the study by Jeremic *et al*., concurrent CRT was associated with a higher rate of Grade 3 or higher levels of leukopenia, neutropenia, and thrombocytopenia.

In our study, the major adverse events in the concurrent CRT group were hematological toxicities, including decreased white blood cell and neutrophil counts. Gastrointestinal toxicities, including anorexia, nausea, vomiting, and constipation, were relatively mild. However, Grade 3 or higher infection and pneumothorax occurred in three (15.8%) and two (10.5%) patients, respectively. Moreover, there was one treatment-related death. The main adverse events in the sequential CRT group were hematologic toxicities, including decreased white blood cell counts; however, there were significantly fewer cases showing Grade 3 or higher white blood cell decreases, a relatively small proportion of other hematologic and non-hematologic toxicities, including hematocytopenia, and one treatment-related death. In this study, the incidence of Grade 3 or higher adverse events was also higher in the concurrent CRT group than that in the sequential CRT group, except for pneumonitis. Despite prophylactic administration of G-CSF in 27 of 32 patients (84.3%), more than 70% of the total patient, 78.9% of patients in the concurrent CRT group, and 61.5% of patients in the sequential CRT group had Grade 3 or higher neutrophil count decreased. All patients who received G-CSF administered it prophylactically during chemotherapy and not during radiotherapy. Neutrophil count decrease occurred at high rate, but febrile neutropenia occurred in 9.4% of overall patients. Although routine prophylactic administration of G-CSF is not usually recommended, clinical guidelines recommend that patients with risk factors for febrile neutropenia who are treated with chemotherapeutic regimens associated with a ≥ 20% risk of febrile neutropenia should be administered G-CSF as primary prophylaxis.^28,29^ Routine prophylactic administration of G-CSF during chemotherapy in CRT in older patients with LD-SCLC is not recommended. However, one report suggests considering primary prophylaxis with G-CSF to prevent febrile neutropenia in male patients with SCLC who are treated with platinum plus etoposide and have a history of radiation therapy, which is a risk factor for febrile neutropenia.^30^ Primary prophylaxis with G-CSF may be considered aggressively during chemotherapy in certain situations.

However, it should be noted that when evaluating toxicity, the criteria for determining the Grade of adverse events may not be consistent across different studies. Table 5 shows that treatment discontinuation mainly occurred owing to failure to complete chemotherapy. In studies evaluating concurrent CRT and this study, the proportion of patients who did not complete treatment was > 30%. In this study, of the 19 patients who received concurrent CRT, 6 did not complete a full cycle of chemotherapy owing to toxicity and 1 discontinued TRT. Of the 13 patients who received sequential CRT, one did not complete a full cycle of chemotherapy owing to toxicity. Regarding treatment completion, the concurrent CRT group had a higher rate of toxicity discontinuation than the sequential CRT group in this study. We speculate that patients in good general condition were treated with concurrent CRT and frail patients were treated with sequential CRT. Therefore, as our findings suggest, it may not be possible to perform concurrent CRT in all older patients (> 75 years) with LD-SCLC. Furthermore, radiation pneumonitis should be considered with caution, as Grade 3 or higher severe pneumonitis occurred in 2/32 patients (6.3%) in our study. To reduce the frequency and severity of radiation pneumonitis, it may be appropriate to set the irradiation field according to the tumor volume after the induction of chemotherapy in the case of sequential CRT.^31^

Older patients with good PS and normal organ function, including those with extensive SCLC, tend to be treated using the same regimens as younger patients undergoing chemotherapy. However, some studies have suggested that these older patients may be at greater risk of severe toxicity as compared to their younger counterparts.^4,32^ Regarding whether chemotherapy regimens based on cisplatin or carboplatin, in combination with TRT, are superior, a meta-analysis demonstrated that both cisplatin-based and carboplatin-based chemotherapy regimens are equally effective in SCLC.^33^ In addition, the studies shown in Table 5 have shown that carboplatin-based combination regimens are relatively more common in older patients. Thus, non-cisplatin chemotherapeutic regimens such as carboplatin and etoposide have become the favored chemotherapy regimens for older patients with SCLC.^34^

Our study population included patients enrolled from 2007–2021, during which time improvements in supportive care, such as antiemetic drugs, and developments in radiation methods and devices may have affected the efficacy and safety of the treatment. As shown in Table 4, approximately half of the patients who relapsed received subsequent chemotherapy. Kasahara *et al*. reported that treatments administered after first-line CRT might affect OS.^35^ In this study, two patients were treated with chemotherapy combined with immune checkpoint inhibitors (ICIs), which may have affected OS. In the future, the use of more active ICIs in older patients with LD-SCLC who relapse after CRT may have a significant impact on the long-term prognosis.

This study had some limitations. First, this study had a retrospective design and a small sample size, thereby limiting the generalizability of the findings. A retrospective study design depends on subjective physician examinations, leading to variabilities in tumor response and PFS data. Second, the intervals between lesion evaluations in this study were not as consistent as those in a prospective trial. Thus, the potential significance of the sources of bias must be considered when interpreting our data. In particular, the severity of non-hematological adverse events may have been underestimated owing to the retrospective nature of this study. Third, patients were treated as inpatients for most of the treatment duration, and data on treatment toxicities were recorded in detail in the patients’ medical records. This exploratory analysis could not be considered definitive. Nevertheless, because it is difficult to collect data on a large number of older patients with LD-SCLC who have received CRT, our findings may be helpful for physicians in determining the optimal treatment choice for this patient group.

In summary, although hematological toxicities, particularly decreased neutrophil counts, were severe, CRT showed favorable efficacy not only in the concurrent CRT group, but also in the sequential CRT group. However, concurrent CRT may not be feasible for all older patients with LD-SCLC, and sequential CRT should be considered as a treatment choice for this patient group. Further prospective trials are warranted to develop and evaluate optimal treatment strategies for older patients with LD-SCLC.

## Supplementary Material

Supplementary Material Details
