# Supplementary material for: A retrospective evaluation of therapeutic efficacy and safety of chemoradiotherapy in older patients (aged ≥ 75 years) with limited-disease small cell lung cancer: insights from two institutions and review of the literature
Source: Radiol Oncol. 2024 Sep 15;58(3):432–43. doi: 10.2478/raon-2024-0054 (PMC11406904; doi:10.2478/raon-2024-0054)
Supplement: Supplementary file 1 — Supplementary Material Details [file raon-2024-0054-sm.pdf]

A retrospective evaluation of therapeutic efficacy and safety of chemoradiotherapy in older patients (aged ≥ 75 years) with limited-disease small cell lung cancer: insights from two institutions and review of the literature

Ayako Shiono, Hisao Imai, Satoshi Endo, Kazunori Katayama, Hideaki Sato, Kosuke Hashimoto, Yu Miura, Shohei Okazaki, Takanori Abe, Atsuto Mouri, Kyoichi Kaira, Ken Masubuchi, Kunihiko Kobayashi, Koichi Minato, Shingo Kato, Hiroshi Kagamu

doi: 10.2478/raon-2024-0054

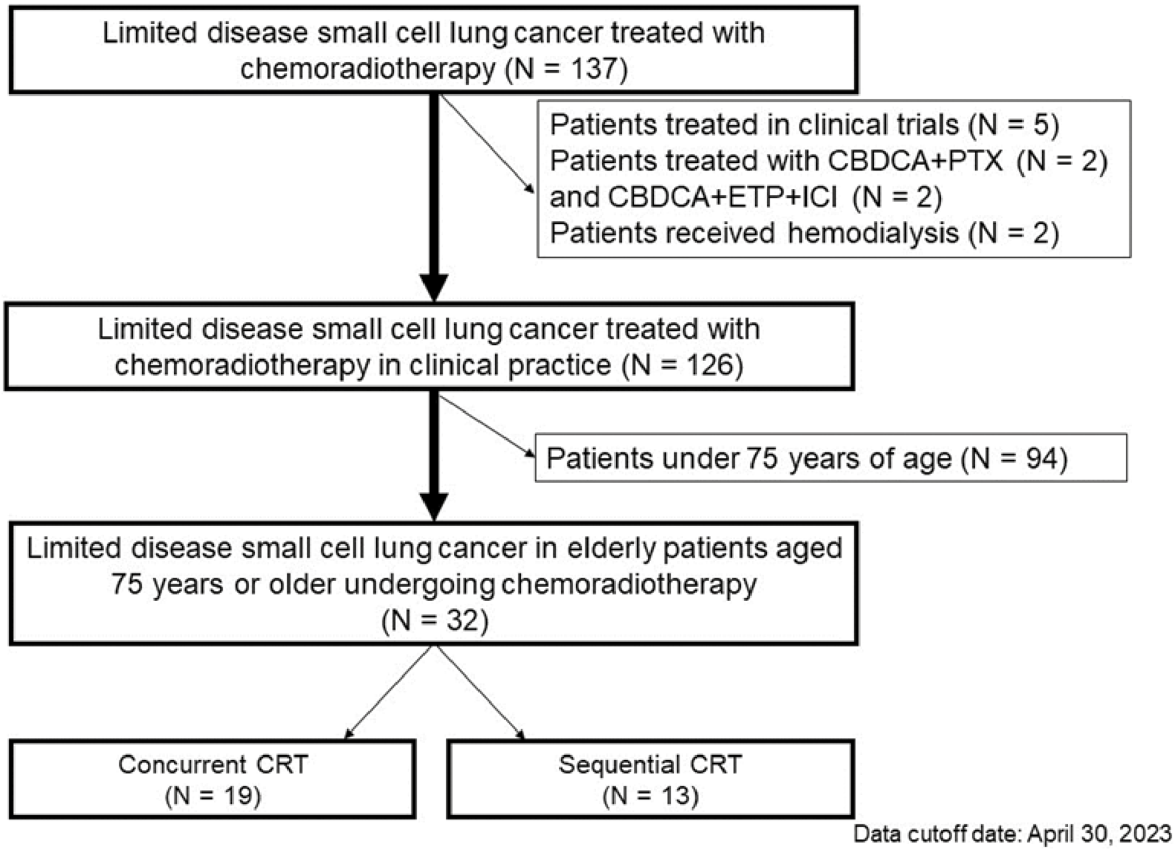

SUPPLEMENTARY FIGURE 1. A flow diagram of patient selection. Patients treated with chemoradiotherapy between April 2007 and June 2021 were eligible for the study.

CBDCA = carboplatin; CRT = chemoradiotherapy; ETP = etoposide; ICI = immune checkpoint inhibitor; PTX = paclitaxel

SUPPLEMENT TABLE 1. Chemotherapeutic treatment delivery

	Total (*N*=32)	Concurrent CRT (*n*=19)	Sequential CRT (*n*=13)
**Chemotherapy regimen**			
**CBDCA + etoposide**	**28**	**16**	**12**
CBDCA (AUC 5) + etoposide (100 mg/ m^2)	7	5	2
CBDCA (AUC 5) + etoposide (80–99 mg/ m^2)	5	1	4
CBDCA (AUC 4) + etoposide (100 mg/ m^2)	1	1	0
CBDCA (AUC 4) + etoposide (80–99 mg/ m^2)	12	9	3
CBDCA (AUC 3) + etoposide (<80 mg/ m^2)	3	0	3
**CDDP + etoposide**	**4**	**3**	**1**
CDDP (80 mg/ m^2) + etoposide (100 mg/ m^2)	1	0	1
CDDP (70 mg/ m^2) + etoposide (100 mg/ m^2)	1	1	0
CDDP (60 mg/ m^2) + etoposide (100 mg/ m^2)	2	2	0

AUC = area under curve; CBDCA = carboplatin: ; CDDP = cisplatin; CRT = chemoradiotherapy
